# Physiological and Pathological Regulation of Peripheral Metabolism by Gut-Peptide Hormones in *Drosophila*

**DOI:** 10.3389/fphys.2020.577717

**Published:** 2020-09-29

**Authors:** Xiaoya Zhou, Guangming Ding, Jiaying Li, Xiaoxiang Xiang, Elisabeth Rushworth, Wei Song

**Affiliations:** ^1^Department of Oncology, Renmin Hospital of Wuhan University, Wuhan, China; ^2^Frontier Science Center for Immunology and Metabolism, Medical Research Institute, Wuhan University, Wuhan, China

**Keywords:** gut hormone, *Drosophila*, metabolism and endocrinology, disease model, nutrient sensing, gut bacteria, tumor-induced wasting, stress sensing

## Abstract

The gastrointestinal (GI) tract in both vertebrates and invertebrates is now recognized as a major source of signals modulating, *via* gut-peptide hormones, the metabolic activities of peripheral organs, and carbo-lipid balance. Key advances in the understanding of metabolic functions of gut-peptide hormones and their mediated interorgan communication have been made using *Drosophila* as a model organism, given its powerful genetic tools and conserved metabolic regulation. Here, we summarize recent studies exploring peptide hormones that are involved in the communication between the midgut and other peripheral organs/tissues during feeding conditions. We also highlight the emerging impacts of fly gut-peptide hormones on stress sensing and carbo-lipid metabolism in various disease models, such as energy overload, pathogen infection, and tumor progression. Due to the functional similarity of intestine and its derived peptide hormones between *Drosophila* and mammals, it can be anticipated that findings obtained in the fly system will have important implications for the understanding of human physiology and pathology.

## Introduction

**Figure 1:**
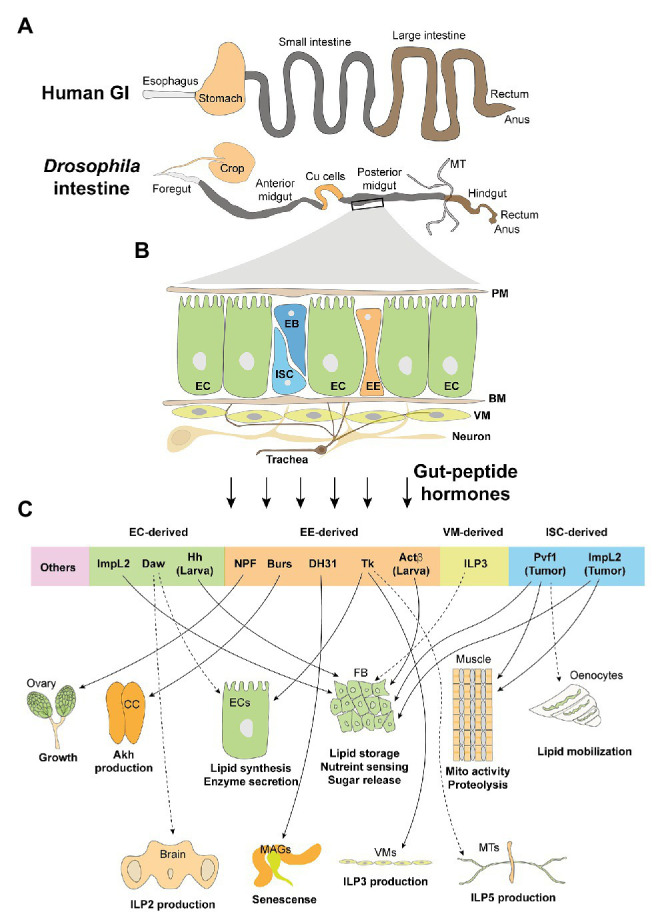
The adult intestine and its cell types and derived peptide hormones. **(A)** Human and *Drosophila* adult intestines share high similarities in the esophagus/foregut, small intestine/midgut, large intestine/hindgut, rectum, as well as anus. Differences include the presence of *Drosophila* Malpighian tubules (MT) that functions as human kidney. Moreover, crop and Cu cells function as storage digestive organs, respectively, to partially mimic the stomach. **(B)** General composition and cell types of adult midgut: PM, peritrophic membrane; EC, enterocyte; ISC, intestinal stem cell; EB, enteroblast; EE, enteroendocrine cell; BM, basement membrane; VM, visceral muscle; neuron and trachea. **(C)** Gut hormones, which are derived by different cell types, target various organs and modulate their metabolic activities. CC, corpora cardiaca; FB, fat body; MAGs, male accessory glands.

More than 100 bioactive gut-peptide hormones are produced by enteroendocrine cells (EEs) in the gastrointestinal (GI) tract, which is thus considered as the biggest endocrine organ in vertebrates (Sun et al., 2018). Emerging therapies, which are based on gut-peptide hormones and have been proven to be efficient in the treatment of metabolic disorders (Alexiadou et al., 2019), have drawn increasing attention to the gut-peptide-hormone modulation of systemic energy balance, including carbo-lipid metabolism in the liver, adipose tissue, muscle, heart, kidney, pancreas, bone, immune cells, as well as brain (Martin et al., 2019). The *Drosophila* intestine exhibits high similarities with the mammalian GI tract, not only in structure and physiology (Figure 1), but also in the production of gut-peptide hormones and its impact on metabolism homeostasis (Miguel-Aliaga et al., 2018). Research using the *Drosophila* system has addressed several fundamental issues regarding the function and regulation of gut-peptide hormones. In this review, we will summarize the types, metabolic impacts, stress sensing, and participating signaling pathways of adult fly gut-peptide hormones under both physiological and pathological conditions.

## Types and Functions of *Drosophila* Gut-Peptide Hormones

The luminal surface of the *Drosophila* adult intestine comprises four cell types (Figure 1B): intestinal stem cells (ISCs), enteroblasts (EBs), enterocytes (ECs), and enterendocrine cells (EEs). The epithelium is covered and protected by a peritrophic membrane (PM), equivalent to human mucus, from intestinal microbes. The epithelial monolayer is aligned on its basal side on the basement membrane (BM). There are visceral muscles (VMs) that drive peristaltic movements, trachea that provide oxygen, and innervated neurons, underneath the BM (Miguel-Aliaga et al., 2018). Similar to that of vertebrates, the majority of gut-peptide hormones are produced by the fly EEs to target distal organs (Reiher et al., 2011; Liu and Jin, 2017). Interestingly, growing evidence indicates that other cell types, like ECs and VMs, also produce bioactive peptides in response to extracellular stresses, many of which have shown systemic metabolic influences and are considered as novel gut-peptide hormones (Figure 1C).

### EE-Derived-Peptide Hormones

A few established proproteins, like allatostatin A (AstA), AstB/Mip, AstC, neuropeptide F (NPF), short neuropeptide F (sNPF), tachykinin (TK), diuretic hormone 31 (DH31), and CCHamides 1 (CCHa1) and CCHa2, which can be processed into over 20 mature peptides, are produced by both larval and adult EEs as shown using antibody detection and proteomic analysis (Veenstra et al., 2008; Veenstra, 2009; Reiher et al., 2011). The protein maturation of the prohormones into multiple bioactive peptides in EEs is processed by a conserved prohormone convertase Amon and, probably, other putative enzyme homologs, like dCPD, Phm, and Pal1/2 (Reiher et al., 2011). Emerging single-cell RNAseq (scRNAseq) technologies have identified additional gut-peptide hormones in the EEs (Guo et al., 2019; Hung et al., 2020; Table 1). Even though several gut-peptide hormones such as DH31 and Tk were previously shown by *in vitro* assays to stimulate gut mobility and possible nutrient delivery decades ago (Siviter et al., 2000; LaJeunesse et al., 2010), the investigation of their physiological roles is largely hampered due to lack of genetic tools. As most Gal4 lines for genes that encode gut-peptide hormones in EEs also target those neurons expressing the same genes in the brain, it is very difficult to distinguish their roles in the gut and brain.

**Table 1:** Summary of metabolic impacts of gut-peptide hormones in *Drosophila*.

Cell types	Gut hormones	Metabolic impacts	Targeted organs
EC	Daw	Decrease EC enzyme production and increase ILP2 production in IPCs (Chng et al., 2014; Ghosh and O’Connor, 2014)	ECs, IPCs
	ImpL2	Decrease lipid storage (Hang et al., 2014)	ECs, FB
	Hh (larva)	Increase lipid mobilization (Rodenfels et al., 2014; Zhang et al., 2020)	FB
EE	Tk	Increase lipid production in ECs and ILP3 release in VMs (Song et al., 2014; Kamareddine et al., 2018)	ECs, VMs
	NPF	Unknown (Ameku et al., 2018)	Ovary
	DH31	Unknown (Takeda et al., 2018)	MAGs
	Burs	Increase Akh release and lipid mobilization (Scopelliti et al., 2019)	Neuron
	Actβ (larva)	Increase Akh response (Song et al., 2017)	FB
ISC/tumor	ImpL2	Increase lipid loss, glycemic level, and muscle wasting (Kwon et al., 2015)	Multiple
	Pvf1	Increase lipid loss and muscle wasting (Ghosh et al., 2019; Song et al., 2019)	FB, muscle, oenocytes
VM	ILP3	Increase systemic insulin signaling and lipid storage (Kamareddine et al., 2018)	Multiple

We have established a *Tk-g-Gal4*, that is predominantly expressed in all Tk^+^ EEs, the most abundant one accounting for ~40% EEs in the midgut (Song et al., 2014), and a very small portion of Tk^+^ neurons in the brain. Using this *Tk-g-Gal4*, we are able to ablate Tk^+^ EEs and diminish Tk production in the gut with rarely affecting its expression in the brain, thus revealing *in vivo* metabolic roles of gut Tk in intestinal and systemic lipid metabolism. Mature gut-peptides Tk1–Tk5, which are processed from the pro-Tk, target the G-protein coupled receptor (GPCR) TkR99D in the ECs and triggers cAMP/PKA signaling to suppress the activity of Sterol regulatory-element binding protein (SREBP) and lipogenic programs in the gut, leading to decreased lipid production in the ECs and reduced lipid storage in the whole body. In addition, gut Tk6, another pro-Tk-derived mature peptide, activates another GPCR TkR86C in the VMs and increases ILP3 production to modulate both local and systemic insulin signaling and lipid homeostasis (Poels et al., 2009; Amcheslavsky et al., 2014; Kamareddine et al., 2018). Tk has been reported to activate Malpighian tubules (MTs; Soderberg et al., 2011), as well as ILP-producing cells (IPCs) in the brain (Birse et al., 2011), *via* TkR99D to regulate ILPs secretion and nutrient-deprivation response as well. Thus, it is believed that gut-derived Tk targets multiple tissues/organs to collectively regulate systemic energy homeostasis.

Single-cell RNAseq and biochemical analysis indicate that Tk^+^ EEs also produce DH31, Burs, NPF, Gpb5, and Nplp2, in different regions of the midgut (Chen et al., 2016a; Guo et al., 2019; Hung et al., 2020). Another study using the *Tk-g-Gal4* revealed that gut NPF non-autonomously controls mating-induced proliferation of germline stem cells *via* ovarian NPF receptor (NPFR; Ameku et al., 2018). Like TkR99D, NPFR is also expressed in MTs and is highly associated with nutrient-deprivation response (Chintapalli et al., 2012). It will be interesting to determine whether gut NPF regulates systemic metabolic homeostasis together with Tk.

*Pros-Gal4*, a driver that specifically targets all EEs in the midgut and a few neuroendocrine cells (Scopelliti et al., 2019), has recently been used for characterizing the functions of gut-peptide hormones in *Drosophila*. Even though the gut-peptide hormone Burs is produced by a small subset of Tk^+^ EEs (Chen et al., 2016a), a group has characterized its opposite role to that of Tk in lipid metabolic control by knocking down its expression in EEs with *Pros-Gal4* (Scopelliti et al., 2019). They demonstrated that Burs remotely activates the GPCR DLgr2 in a specialized type of neurons and subsequently inhibits the activity of neighbor neuroendocrine cells that produce adipokinectic hormone (Akh) in the corpora cardiaca (CC). Akh is the key hormone modulating lipid mobilization and carbohydrate release in the fat body *via* AkhR/cAMP/PKA cascade and promoting food seeking through a subset of neurons in the brain (Kim and Rulifson, 2004; Gronke et al., 2007; Huang et al., 2020). Gut-derived Burs, therefore, impairs Akh-associated triglyceride (TAG) breakdown and promotes systemic lipid accumulation.

Using *Pros-Gal4* to eliminate the expression of DH31 in the midgut, another group revealed that gut-derived DH31 remotely regulates the senescent responses of male accessory glands (MAGs; Takeda et al., 2018), suggesting the hormonal effects of this gut peptide. Since DH31 has been reported to target MTs *via* the GPCR Dh31-R (Coast et al., 2001; Johnson et al., 2005), it could be anticipated that gut-derived DH31 also modulates MTs function, like water flux as well as systemic metabolic homeostasis.

Earlier studies indicated that AstC^+^ EEs, another large subpopulation of EEs in the midgut, also produce multiple gut peptides, such as AstA, AstB/Mip, CCHa1, and CCHa2 (Chen et al., 2016a; Guo et al., 2019). CCHa2 deficiency is associated with impaired ILPs production in larval brain (Ren et al., 2015; Sano et al., 2015). Even though, at least, fat-body-produced CCHa2 is shown to contribute to the regulation of ILP2 secretion (Sano et al., 2015), the origins of bioactive CCHa2 are still controversial. Using different drivers to examine whether brain‐ or gut-derived CCHa2 also targets adult IPCs in the brain and modulate systemic energy balance will help address this puzzle in the future (Ren et al., 2015; Sano et al., 2015).

Global *AstA* removal results in disruption of systemic lipid homeostasis and appetite control (Hergarden et al., 2012; Chen et al., 2016b). It is considered as a direct regulation of Akh and insulin releases by AstA, as the GPCR of AstA, AstA-R2, is highly expressed in both Akh‐ and insulin-producing cells (Hentze et al., 2015). However, these studies failed to distinguish the differential functions between gut‐ and brain-derived AstA. Another group used *Pros-Gal4* to knock down AstA expression in the midgut and revealed its systemic role as a peptide hormone in longevity modulation (Takeda et al., 2018). Therefore, it is possible that gut AstA modulates release of Akh or insulin to affect energy homeostasis, but further genetic validation is required.

Remarkably, flies lacking all EEs are relatively normal in terms of food intake and carbo-lipid metabolism but are shorter-lived (Amcheslavsky et al., 2014), indicating anticipated counter-regulatory impacts between distinct gut-peptide hormones on energy balance. We speculate that flies might execute differential metabolic impacts *via* certain gut-peptide hormones in the context of various stress responses.

In addition to adult EEs, larval EEs produce peptide hormones to regulate systemic metabolism as well. We have recently characterized Activin-β (Actβ) as an important EE-derived peptide hormone in the larval midgut (Song et al., 2017). Using *Actβ-Gal4*, *Tk-g-Gal4*, as well as *Pros-Gal4*, we uncovered that gut-derived Actβ remotely activates Babo/Smox signaling in the fat body and enhances its Akh response, leading to carbohydrate breakdown and elevation of glycemic level.

### EC-Derived-Peptide Hormones

Enterocyte is the biggest cell population in both larval and adult midguts and also produces multiple peptide hormones that regulate systemic energy homeostasis (Figure 1C and Table 1). ImpL2, an established hormone robustly blocking circulating ILP bioavailabilities and downregulating systemic insulin signaling (Honegger et al., 2008), is majorly expressed in the adult ECs (Hung et al., 2020) and is associated with the impairment of lipid metabolism caused by gut bacteria (Hang et al., 2014). Adult ECs also produce the TGF-beta/activin ligand, Dawdle (Daw), into the hemolymph to impair systemic carbo-lipid homeostasis. Possible mechanisms include that Babo/Smox signaling modulates the expression of carbohydrases and insulin secretion in the ECs and brain IPCs, respectively (Chng et al., 2014; Ghosh and O’Connor, 2014). In addition, larval ECs secret Hedgehog (Hh), a conserved ligand that regulates metabolism and development across species (Teperino et al., 2014), *via* lipoprotein particles into the hemolymph to directly activate Ci/Bmm-axis and lipolysis in the fat body, resulting in systemic lipid loss (Rodenfels et al., 2014; Zhang et al., 2020).

There are a few cytokines, like Upd3 and Dpp (Jiang et al., 2009; Tian and Jiang, 2017), produced by ECs to maintain local tissue homeostasis. ECs also secret bioactive enzymes, including multiple trypsins for food digestion and peptidoglycan recognition proteins (PGRPs) that degrade gut-bacteria-derived peptidoglycans (PGNs) and blunt inflammatory responses (Guo et al., 2014; Charroux et al., 2018), to affect systemic carbo-lipid metabolism. It will be interesting to investigate whether they function as circulating hormones and directly affect metabolic activities of other organs in the future.

### Peptide Hormones Derived by Other Intestinal Cell Types

Other gut cell types also produce peptide hormones that execute important metabolic roles (Figure 1C and Table 1). VM-secreted ILP3, which was previously characterized to maintain local insulin signaling and ISC activity in the gut (O’Brien et al., 2011), is currently found to contribute to insulin signaling in the whole fly (Kamareddine et al., 2018). ISCs that bear an active oncogene *yki* proliferate as malignant tumors and produce large amounts of bioactive peptide hormones, like ImpL2 and Pvf1, to impair lipid metabolism in the fat body (Kwon et al., 2015; Song et al., 2019). Despite the peptide hormones mentioned above, other cytokines/peptides, such as Delta (Dl) in ISCs (Ohlstein and Spradling, 2007), vein (vn) in VMs (Biteau and Jasper, 2011), PDF in neurons (Talsma et al., 2012), as well as Dpp in gut-associated trachea and hemocytes (Li et al., 2013; Ayyaz et al., 2015; Table 2), that influence ISC activity and local gut homeostasis have shown very limited impacts on metabolic activities of distal organs. Thus, we will not discuss them further in this review.

**Table 2:** Summary of gut-produced cytokines/peptides in *Drosophila*.

Cell types	Gut peptides
ISC/EB	Dl, ILP6, egr, Hh, spi, wg, Upd1, and Upd2 (Ohlstein and Spradling, 2007; Jiang et al., 2009; Karpowicz et al., 2010; Osman et al., 2012; Tian et al., 2015, 2016; Doupe et al., 2018)
EC	Hh, Dpp, Gbb, Krn, PGRP-SA, PGRP-SB1, PGRP-SC1a, PGRP-SC1b, PGRP-SC2, PGRP-SD, PGRP-LB, Upd1, Upd2, and Upd3 (Werner et al., 2000; Bischoff et al., 2006; Jiang et al., 2009, 2011; Osman et al., 2012; Guo et al., 2014; Tian and Jiang, 2014, 2017; Lee et al., 2015; Iatsenko et al., 2016)
EE	AstA, AstC, AstB/Mip, CCHa1, CCHa2, sli, Orcokinin B, CCAP, Nplp2, Gbp5, and ITP (Veenstra et al., 2008; Biteau and Jasper, 2014; Veenstra and Ida, 2014; Ren et al., 2015; Chen et al., 2016a; Benguettat et al., 2018; Rommelaere et al., 2019; Hung et al., 2020)
Hemyocyte	Dpp (Ayyaz et al., 2015)
Neuron	sNPF, DH44, PDF, and AstA (Talsma et al., 2012; Dus et al., 2015; Chen et al., 2016b; Shen et al., 2016)
Trachea	Dpp (Li et al., 2013)
VM	Vn, wg, and Dpp (Jiang and Edgar, 2009; Tian et al., 2016; Tian and Jiang, 2017)

## Dietary Regulation of Gut-Peptide-Hormone Production

Gut-peptide hormones act in concert to modulate the physiologies of both GI itself and other distal organs to ensure nutrient absorption, delivery, mobilization, as well as storage, after a meal. Note that, the production and release of gut-peptide hormones are directly controlled by the digested food in a feedback loop. For example, Tk production in the midgut is suppressed (Song et al., 2014), while Burs production is enhanced (Scopelliti et al., 2019), under the feeding condition. On the other hand, chronic high-caloric diets also perturb the production of gut-peptide hormones and systemic metabolic balance. Advanced imaging tools in *Drosophila* have demonstrated and visualized the *in vivo* nutrient sensing, which is associated with the production of gut peptides, in the midgut in response to individual component(s) in the food.

### Amino Acids

A recent study using the cytoplasmic calcium reporter CaLexA has indicated that dietary amino acids like casein peptone and lysine directly activate intracellular Ca^2+^ cascade, an increase in which is associated with peptide release in multiple endocrine cells (Dus et al., 2015; Benguettat et al., 2018; Oh et al., 2019), in Tk^+^ and DH31^+^ EEs (Park et al., 2016). Consistently, the protein level and release of Tk peptides in EEs are increased in starved flies and other insects to control gut mobility and EC lipogenesis (Winther and Nassel, 2001; Song et al., 2014), while refeeding flies only yeast that contains plenty of amino acids, but not sucrose or coconut oil, blunts the increase in Tk peptide level (Song et al., 2014). Several transporters or receptors that sense distinct amino acids and trigger the downstream signaling pathways have been characterized in *Drosophila* (Maniere et al., 2020). However, their roles in EEs are not fully identified yet.

### Carbohydrates

Nutrient deprivation reduces release of Burs from EEs into the hemolymph and causes intestinal Burs accumulation. Either sucrose refeeding or diminishing the expression of Glut1, a glucose transporter, in the EEs, alleviates Burs accumulation (Scopelliti et al., 2019), confirming the control of Burs release by dietary glucose or sucrose. In addition, starvation suppresses, while yeast refeeding restores, global larval CCHa2 mRNA levels (Sano et al., 2015). Interesting, the researchers found that glucose, but not amino acids, contained in yeast paste results in CCHa2 transcriptional suppression and further uncovered fat body TOR signaling as the sensor (Sano et al., 2015). It is possible that CCHa2 transcription in the EEs is similarly regulated by nutrients as well, but more biochemical and genetic evidence are required for further validation. In addition to hormone production and release, dietary carbohydrates also influence EE mass in the larval midgut. We have uncovered that chronic high-sucrose diet perturbs larval gut homeostasis and promotes EE differentiation with unknown mechanism(s), resulting in excessive Actβ production, enhanced Akh response in the fat body, and hyperglycemia (Song et al., 2017).

### Lipids

The absorption of fatty acids in fly intestine has not been carefully studied, even though homologs of fatty acid transport proteins (FATPs), fatty acid translocase FAT (CD36), and fatty-acid-binding proteins (FABPs) that regulate fatty acid binding and transport are all present in *Drosophila* (Adams et al., 2000). So far, there is no clear evidence suggesting a direct regulation of gut-peptide hormone release by dietary lipids in *Drosophila*. However, a group demonstrated that adult EE numbers are decreased by lipid-deleted food, while increased by high-cholesterol food (Obniski et al., 2018). They also revealed that dietary cholesterol, absorption of which is regulated by the Hr96/NPC2b axis in the intestine, influences endomembrane lipid composition and the subcellular localization, trafficking, as well as turnover, of the Delta/Notch complex in the ISCs. These changes further suppress Notch signaling in the EBs and promote EE differentiation and subsequent production of EE-derived peptide hormones (Obniski et al., 2018).

### Non-nutrient Components

An interesting study reported that 10 gustatory receptors are expressed in the EEs (Park and Kwon, 2011). These gustatory receptors can be activated by diverse dietary chemicals, such as caffeine, bitter compounds, and carbohydrates (Hanlon and Andrew, 2015; Table 3). Even though the ligand/receptor action in the gut is not characterized yet, it raises the hypothesis that diet might regulate gut-peptide hormone release and subsequent systemic metabolism *via* taste components in addition to nutrients. On the other hand, a recent study demonstrated that yeast particles trigger mechanical stress in the midgut and activate ISC proliferation (Li et al., 2018a). Several mechanical sensors, including TrpA1 and Piezo that activate Ca^2+^ cascade, are reported to be expressed in the adult EEs (Du et al., 2016; He et al., 2018), it will be likely that food containing indigestible particles or fibers would perturb the production and release of gut-peptide hormones independent of nutrients.

**Table 3:** Gustatory receptor expression in adult EEs (Park and Kwon, 2011; Hanlon and Andrew, 2015).

Gustatory receptors	Producing cells	Putative ligands
Gr28a, Gr28b, Gr33a, Gr93a	Tk/NPF^+^ EEs	Caffeine
Gr36c, Gr59a	Tk/NPF^+^ EEs	Bitter compounds
Gr39a	Tk/NPF^+^ EEs	Mating pheromone
Gr43a, Gr64a	Tk/NPF^+^ EEs	Carbohydrates
Gr58c	NPF^−^ EEs	Unknown

## Gut Microbiota and Peptide-Hormone Production

The gut microbiota emerges as a neglected metabolic organ based on a number of important discoveries of its products, including short-chain fatty acids (SCFAs), amino acids, and bacteriocin, that regulate host immunity and metabolism (Depetris-Chauvin et al., 2017; Li et al., 2018b; Qiao et al., 2019). The simpler microbiota and signaling systems of the *Drosophila* have provided researchers with a unique opportunity to study the impact of either commensal or pathogenic intestinal microbes on host feeding behavior and energy balance in a more controlled and targeted fashion (Capo et al., 2019). Several studies further illustrated gut-peptide hormones as key regulators mediating host immune response and carbo-lipid metabolism in response to diverse gut microbiota.

### Commensal Bacteria

**Figure 2:**
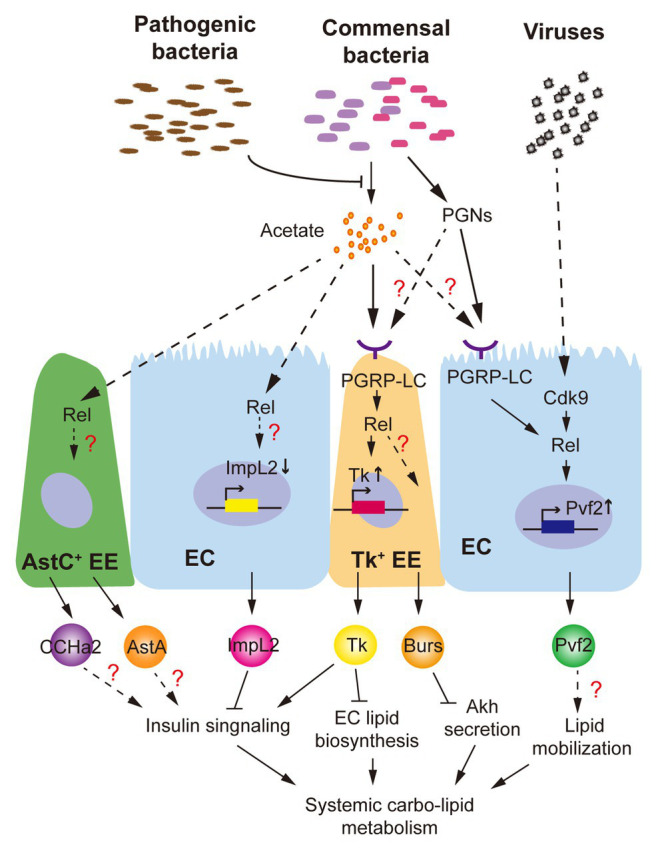
*Drosophila* intestinal microbiota influence production of gut-peptide hormones. Commensal bacteria-derived acetate, which is suppressed by *Vibrio cholerae*, a pathogenic bacterium, promotes Tk production in enterocytes (ECs) *via* activation of PGPR-LC/Rel signaling and modulates systemic insulin signaling and lipid homeostasis. Production of other gut hormones such as ImpL2, CCHamides 2 (CCHa2), allatostatin A (AstA), and Burs regarding systemic energy balance is probably regulated by similar mechanism(s). Viral infection and commensal bacteria-derived peptidoglycans (PGNs) activate Cdk9/Rel and PGRP-LC/Rel cascades, respectively, to increase production of Pvf2, which is associated with Pvr activation in the fat body and lipid loss.

Different groups have shown that axenic adult flies exhibit a delayed development, lipid and glycogen accumulation, and hyperglycemia (Shin et al., 2011; Wong et al., 2014). Impairment of systemic insulin signaling, which is associated with the dominant gut commensal microbiota (*Lactobacillus plantarum* and *Acetobacter pomorum*), is considered as a major regulator. *L. plantarum* produces branched-chain amino acids to directly activate host TOR and insulin signaling (Storelli et al., 2011), while SCFAs like acetate produced by *A. pomorum* modulate systemic insulin signaling *via* gut-peptide hormone production (Kamareddine et al., 2018). As we mentioned, both mRNA and peptide levels of gut Tk, which promotes ILP3 production in VMs *via* TkR99D activation to modulate systemic insulin signaling and energy balance (Poels et al., 2009), are increased by intestinal acetate (Kamareddine et al., 2018). Even though the receptors sensing acetate are not yet identified in *Drosophila*, researchers indicated that intestinal microbial acetate activates immune responses *via* PGRP-LC/Rel signaling to increase Tk^+^ EEs mass and Tk synthesis in the gut (Figure 2). Note that, acetate-activated Rel signaling is observed not only in Tk^+^ EEs but also in AstC^+^ EEs, ISCs, and ECs (Kamareddine et al., 2018). EC-derived ImpL2 is previously reported to be suppressed by intestinal acetate content (Hang et al., 2014). Further, other hormones, like AstA and CCHa2 produced in AstC^+^ EEs, are probably associated with ILP2 production and systemic insulin response as well (Hentze et al., 2015; Ren et al., 2015). Therefore, it is quite likely that intestinal acetate modulates differential gut-peptide hormone production in multiple cell types *via* Rel activation and orchestrates their influences on insulin signaling and host metabolism (Figure 2).

**Figure 3:**
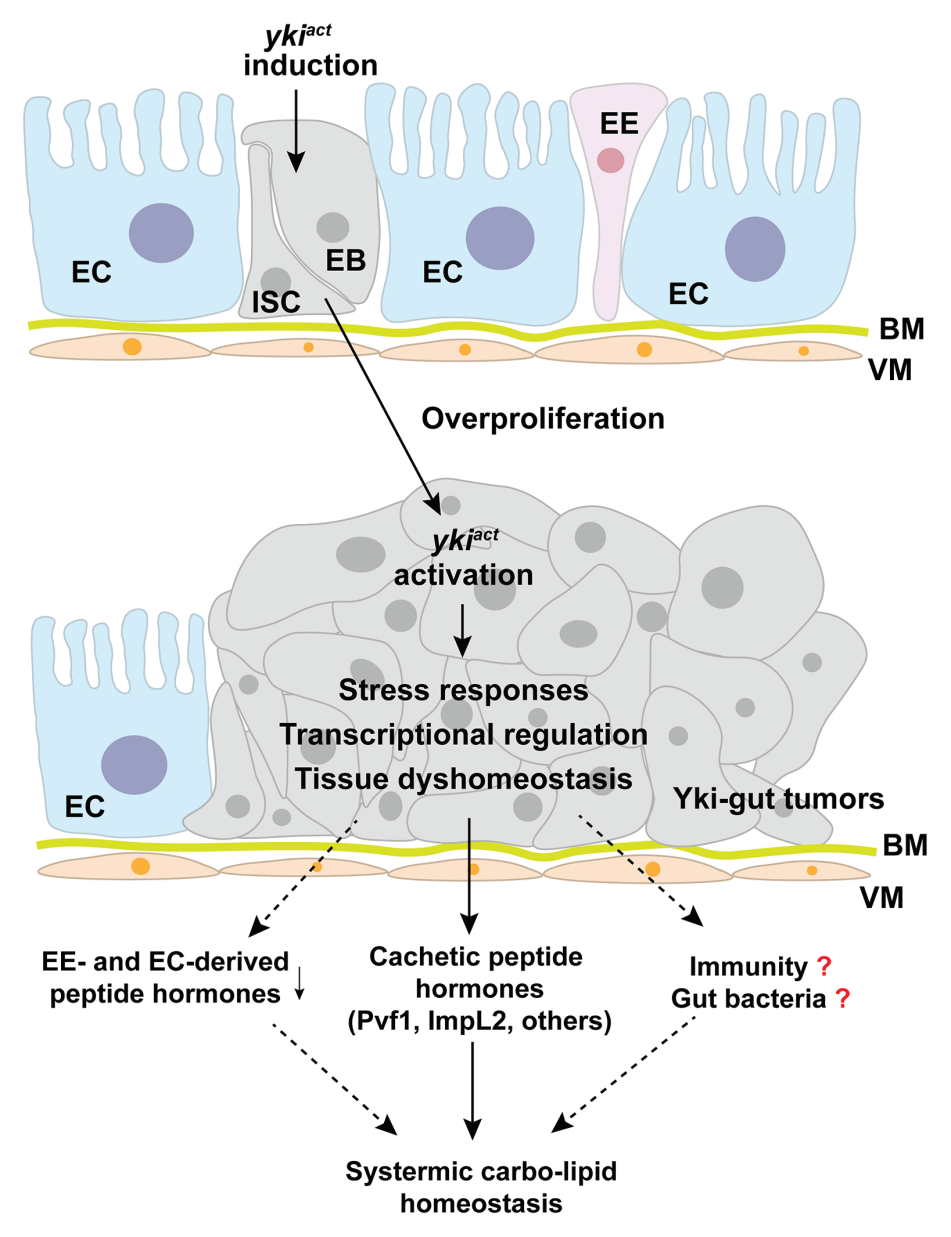
Yki-tumor-bearing gut produces cachectic peptide hormones. Induction of an active *yki* in the intestinal stem cells (ISCs) results in malignant tumor-cell growth in the midgut and production of large amounts of cachectic peptide hormones, including ImpL2, Pvf1, and others. Potential molecular mechanisms include *yki*-associated transcriptional regulation, stress responses, tissue dyshomeostasis, and subsequent immune regulation. EC, enterocyte; ISC, intestinal stem cell; EB, enteroblast; EE, enteroendocrine cell; BM, basement membrane; VM, visceral muscle.

### Pathogenic Bacteria

A few studies have demonstrated that pathogenic bacteria also perturb host metabolism through gut-peptide hormones. Oral infection of *Vibrio cholerae*, a life-threatening bacterium for both human and *Drosophila*, does not influence the gut microbiota or the epithelial barrier but reprograms acetate metabolism in the gut through acetate metabolic genes, *CrbR* and *CrbS* (Hang et al., 2014). In response to intestinal microbial acetate switch, production of ImpL2 and Tk is affected to impair systemic insulin signaling (Hang et al., 2014; Kamareddine et al., 2018). Dietary acetate supplementation further successfully alleviates *Vibrio cholerae*-disrupted host insulin signaling and metabolism. Note that, PGRP-LC/Rel signaling, which is shown to regulate Tk production, could be modulated by various PGNs derived by non-commensal bacteria as well (Royet and Charroux, 2013). Another group recently showed that septic, but not oral, infection of bacterial pathogen *Photorhabdus luminescens*, *Photorhabdus asymbiotica*, or non-pathogenic *Escherichia coli* modulate gut Tk production without affecting gut microbiota homeostasis, resulting in lipid accumulation in the gut and whole body (Harsh et al., 2019). This evidence indicates that gut-peptide hormone could be regulated by the circulating PGNs derived from non-commensal bacteria. Given the vast products such as virulence factors, secreted peptides, and metabolites produced by both commensal and non-commensal bacteria in the fly intestine, it will be necessary to dissect their impacts on gut-peptide hormones in terms of receptors sensing them and the signaling pathways they regulate.

### Viruses

*Drosophila* C virus (DCV), a natural RNA virus for *Drosophila*, has recently been found to cause severe mortality as well as depleted stores of triglycerides and glycogen in adult flies (Arnold et al., 2013; Chtarbanova et al., 2014). The morphology and structure of midgut in DCV-infected flies are severely impaired. Importantly, the production of Pvf2, a peptide homolog of human platelet-derived growth factor (PDGF) and vascular endothelial growth factor (VEGF), is dramatically upregulated in the ECs by the Cdk9/Rel pathway in response to viral infection (Sansone et al., 2015). Although in this study Pvf2 is shown to mediate intestinal antiviral immunity, its metabolic impact is not investigated yet. Interestingly, activation of Pvr, the receptor of Pvf2, in the fat body and oenocytes results in systemic lipid loss (Zheng et al., 2017; Ghosh et al., 2019; Song et al., 2019), phenocopying the DCV infection and suggesting a Pvf2/Pvr axis in the gut-to-fat-body communication. Infection of other RNA viruses like Flock House virus (FHV) also leads to the decline of systemic lipid accumulation (Chtarbanova et al., 2014), whether gut-peptide hormone is involved in FHV-associated metabolic disruption is an interesting question to address in the future.

## Gut Tumors and Peptide-Hormone Production

Tumor-bearing guts have recently been characterized as a differentiated endocrine organ that remotely impairs systemic metabolic homeostasis and causes a cachexia-like phenotype. Induction of an active oncogene *yki*, the homolog of human Yap1, in the ISCs leads to malignant tumorigenesis in the gut and subsequent wasting of host organs, including ovary degeneration, lipid loss, muscle dysfunction, hyperglycemia, as well as mortality (Kwon et al., 2015; Song et al., 2019). Because the affected flies eat normally, it is less likely that yki-gut tumors impair nutrient absorption in the gut. Integrating transcriptome analysis and RNAi screening, we have uncovered that yki-gut tumors produce various peptide hormones to disrupt the balance of systemic anabolism and catabolism (Figure 3).

First, yki-gut tumors release large amounts of ImpL2 to suppress IGF/insulin signaling and its associated anabolism in multiple host organs. As a consequence, ovary size and storages of lipid and glycogen in the fat body are decreased. The flies climb poorly, as the mitochondrial integrity and activity are both impaired in the skeletal muscles (Kwon et al., 2015). Second, yki-gut tumors produce excessive Pvf1, another hormone homologous to mammalian VEGF and PDGF, to extensively activate Pvr/MEK cascade and promote catabolism in the host organs, including lipid and carbohydrate breakdown in the fat body and muscular protein degradation. Small-molecule inhibitors against MEK/ERK strongly alleviate the wasting effects in yki-tumor-bearing flies, as well as C26-tumor cell models, providing pharmaceutical opportunities in prevention and treatment of cancer-associated cachexia (Song et al., 2019). Third, other potential tumor-derived peptide hormones have also been characterized using RNAi screening to regulate host wasting with unknown mechanisms (Song et al., 2019; Figure 3).

How yki activation in ISCs modulates production of cachectic peptide hormones is currently unknown. Possible mechanisms could include yki-induced direct transcriptional regulation of certain peptides and yki-associated ISC proliferation that enlarges the mass of peptide-producing cells. However, the transcriptional levels of these cachectic peptide hormones are increased far more than ISC marker genes and yki-target genes, like *diap1* and *Ex*, in the yki-gut tumors (Song et al., 2019). We, therefore, speculate that yki-activation might also trigger unknown intracellular stress responses to increase peptide-hormone production in a cascade-amplification fashion (Figure 3).

Nevertheless, we also noticed that yki-gut tumors perturb midgut homeostasis by increasing the mass of ISCs but decreasing that of ECs and EEs. As expected, most of the endogenous immune-associated enzymes and peptide hormones that are produced by ECs and EEs are suppressed (Song et al., 2019; Figure 3). Whether these endogenous enzymes, which maintain intestinal bacteria balance and systemic immune response, and peptide hormones contribute to host wasting is another insightful question to be addressed.

## Other Pathological Conditions and Gut-Peptide Hormones

*Drosophila* model has also indicated high associations between systemic metabolism and other pathological conditions. For instance, aged flies exhibit less intestinal and systemic TAG storages (Karpac et al., 2013), while either diet restriction or impaired insulin signaling significantly extends lifespan and increases systemic TAG accumulation (Song et al., 2010; Luis et al., 2016). Recent studies have uncovered important clues regarding participation of gut-peptide hormones, including increased EE mass and bacterial load that are associated with gut-peptide production in aged adult midgut (Ayyaz and Jasper, 2013; He et al., 2018). Moreover, removal of gut-derived AstA and DH31 modulate fly longevity in an opposite manner (Takeda et al., 2018). It will be essential to examine whether gut-peptide hormones are involved in the regulation of aging and carbo-lipid metabolic homeostasis.

Another example is sleep deprivation, a well-established condition that disrupts systemic metabolic balance (Stahl et al., 2017). Researchers have revealed that an EC-derived amino acid, D-serine, is essential for sleep control (Dai et al., 2019), indicating a participating role of fly gut. Strikingly, a recent study further demonstrated that sleep deprivation results in accumulation of reactive oxygen species (ROS) and triggers consequent oxidative stress specifically in the gut, whereas diminishment of ROS accumulation in the gut improves survival without sleep in flies (Vaccaro et al., 2020). Given the fact that ROS regulates diverse metabolic signaling pathways, as well as tissue homeostasis and commensal bacterial control, in the gut (Ha et al., 2005; Ayyaz and Jasper, 2013; Xu et al., 2017), we speculate that ROS-associated production and release of gut-peptide hormone might function as a nexus between sleep and metabolic homeostasis and beyond.

## Conclusion and Outlook

**Figure 4:**
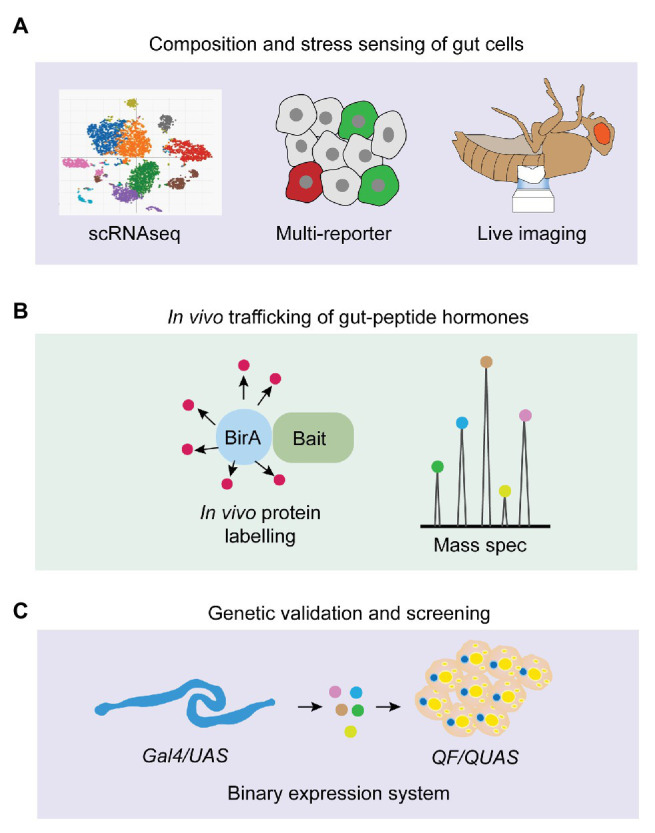
Strategic options for future gut-peptide hormone research in *Drosophila*. **(A)** Single-cell RNAseq (scRNAseq), multi-reporter system, and live imaging can be used to monitor homeostatic composition of intestinal cells and various stress responses that trigger gut-peptide hormone production under different physiological conditions. **(B)** BirA-induced biotin labeling of endogenous protein can be applied to identify novel gut-peptide hormones, investigate their *in-vivo* trafficking, and characterize their targeted organs. **(C)** Binary expression system and genetic screening in *Drosophila* offer us a unique opportunity to investigate peptide-hormone-induced gut-to-other-organ axis and metabolic modulation at the genetic level.

In this review, we have summarized the gut-hormone regulation of systemic metabolism and its essential impacts on physiological and pathogenic outputs, focusing on their genetic characterization, stress-sensing, as well as the mechanisms of act, in *Drosophila*. Despite the gained knowledge and ongoing functional validation of gut-peptide hormones, several fundamental questions in this field still remain unaddressed (Figure 4). In particular, the stress-sensing of intestinal cells regarding peptide production in response to multiple internal and external stimuli is largely unknown. Growing findings of novel stress responses, such as mechanical stress induced by different components in the food (He et al., 2018; Li et al., 2018a), local hypoxic response caused by bacterial infection (Valzania et al., 2018; Krejcova et al., 2019), immune response triggered by intestinal microbial metabolites (Kamareddine et al., 2018), as well as the newly-identified oxidative stress associated with sleep loss (Vaccaro et al., 2020), keep shaping our current understanding of intestinal phenomena. Therefore, integrating multi-reporter system, long-term live imaging (Martin et al., 2018), and scRNAseq to monitor diverse stress responses and study whether and how gut-peptide-hormone production is affected by them will add new dimensions for exploiting gut physiology and metabolic homeostasis (Figure 4A). This strategy will also help illustrate the compositional change of different gut cells that produce distinct hormones and the orchestrating impacts on systemic metabolism under chronic conditions, like tumor progression and high-caloric diet (Figure 4A). *In vivo* trafficking of hormones is difficult to achieve due to limited genetic tools. Recent studies, which engineered a promiscuous biotin ligase, BirA, to specifically label secreted proteins including peptide prohormones (Stevens et al., 2019; Droujinine et al., 2020), are very promising to address the limitation (Figure 4B). They used a fused BirA to biotinylate all proteins in the muscle ER and detected biotin-labeled proteins in the blood to identify potential myokines. Moreover, they further detected biotin-labeled proteins in the other organs to characterize *in vivo* trafficking of these myokines from skeletal muscle to the fat body (Droujinine et al., 2020). Genetic validation is required to confirm the physiological outputs of gut-peptide hormone-induced interorgan communication. The binary expression systems such as LexA/LexAop (Kockel et al., 2016) and QF/QUAS (Riabinina and Potter, 2016) together with Gal4/UAS offer us a unique opportunity. For instance, we could increase the release of a gut-peptide hormone using the *LexA/LexAop* system and simultaneously block its receptor or downstream signaling pathways in the receiving organ using the *Gal4/UAS* system to evaluate the physiological regulation of the particular gut-peptide hormone under study. Conversely, we can also use the *QF/QUAS* system to set up reliable readouts (e.g., Ca^2+^ signaling in Akh-producing cells induced by Burs) and screen for the potential stress pathways and trafficking regulators of the matched hormones (e.g., Burs) with the *Gal4/UAS* system (Figure 4C).

Taken together, the *Drosophila* organism with the accessibility of genetic tools, the simplicity of its genome, and the feasibility for disease modeling, will serve as a powerful system for the future research of gut-peptide hormones. Some of the metabolic regulations that are found in *Drosophila* have recently been shown to be similar in mammals. For example, similar to fly Tk regulation of ILP3/5 secretion, mammalian Substance P and neurokinin A also promote insulin release (Schmidt et al., 2000). Mammalian galanin inhibits insulin secretion as fly AstA does (Tang et al., 2012). Like fly Pvf1, VEGFs are also produced in malignant colon tumors and found to promote lipid mobilization (Sun et al., 2012; Bendardaf et al., 2017). These evidence might, therefore, become relevant in the context of human physiology and pathologies such as diet-induced obesity and diabetes, infectious diseases, and cancer cachexia.

## Author Contributions

XZ wrote the part of bacteria-induced gut hormone production. GD wrote the section of gut hormone types. JL wrote the section of tumor-induced gut hormone production. XX wrote the part of dietary regulation. ER wrote the section of other conditions. WS discussed and organized the whole manuscript. All authors contributed to the article and approved the submitted version.

### Conflict of Interest

The authors declare that the research was conducted in the absence of any commercial or financial relationships that could be construed as a potential conflict of interest.

## References

[ref1] Adams M. D. Celniker S. E. Holt R. A. Evans C. A. Gocayne J. D. Amanatides P. G.. (2000). The genome sequence of *Drosophila melanogaster*. Science 287, 2185–2195. 10.1126/science.287.5461.2185, PMID: 10731132

[ref2] Alexiadou K. Anyiam O. Tan T. (2019). Cracking the combination: gut hormones for the treatment of obesity and diabetes. J. Neuroendocrinol. 31:e12664. 10.1111/jne.12664, PMID: 30466162 PMC6563152

[ref3] Amcheslavsky A. Song W. Li Q. Nie Y. Bragatto I. Ferrandon D.. (2014). Enteroendocrine cells support intestinal stem-cell-mediated homeostasis in *Drosophila*. Cell Rep. 9, 32–39. 10.1016/j.celrep.2014.08.052, PMID: 25263551 PMC4198943

[ref4] Ameku T. Yoshinari Y. Texada M. J. Kondo S. Amezawa K. Yoshizaki G.. (2018). Midgut-derived neuropeptide F controls germline stem cell proliferation in a mating-dependent manner. PLoS Biol. 16:e2005004. 10.1371/journal.pbio.2005004, PMID: 30248087 PMC6152996

[ref5] Arnold P. A. Johnson K. N. White C. R. (2013). Physiological and metabolic consequences of viral infection in *Drosophila melanogaster*. J. Exp. Biol. 216, 3350–3357. 10.1242/jeb.088138, PMID: 23685974

[ref6] Ayyaz A. Jasper H. (2013). Intestinal inflammation and stem cell homeostasis in aging *Drosophila melanogaster*. Front. Cell. Infect. Microbiol. 3:98. 10.3389/fcimb.2013.00098, PMID: 24380076 PMC3863754

[ref7] Ayyaz A. Li H. Jasper H. (2015). Haemocytes control stem cell activity in the *Drosophila* intestine. Nat. Cell Biol. 17, 736–748. 10.1038/ncb3174, PMID: 26005834 PMC4449816

[ref8] Bendardaf R. El-Serafi A. Syrjanen K. Collan Y. Pyrhonen S. (2017). The effect of vascular endothelial growth factor-1 expression on survival of advanced colorectal cancer patients. Libyan J. Med. 12:1290741. 10.1080/19932820.2017.1290741, PMID: 28245709 PMC5345584

[ref9] Benguettat O. Jneid R. Soltys J. Loudhaief R. Brun-Barale A. Osman D.. (2018). The DH31/CGRP enteroendocrine peptide triggers intestinal contractions favoring the elimination of opportunistic bacteria. PLoS Pathog. 14:e1007279. 10.1371/journal.ppat.1007279, PMID: 30180210 PMC6138423

[ref10] Birse R. T. Soderberg J. A. Luo J. Winther A. M. Nassel D. R. (2011). Regulation of insulin-producing cells in the adult *Drosophila* brain via the tachykinin peptide receptor DTKR. J. Exp. Biol. 214, 4201–4208. 10.1242/jeb.062091, PMID: 22116763

[ref11] Bischoff V. Vignal C. Duvic B. Boneca I. G. Hoffmann J. A. Royet J. (2006). Downregulation of the *Drosophila* immune response by peptidoglycan-recognition proteins SC1 and SC2. PLoS Pathog. 2:e14. 10.1371/journal.ppat.0020014, PMID: 16518472 PMC1383489

[ref12] Biteau B. Jasper H. (2011). EGF signaling regulates the proliferation of intestinal stem cells in *Drosophila*. Development 138, 1045–1055. 10.1242/dev.056671, PMID: 21307097 PMC3042864

[ref13] Biteau B. Jasper H. (2014). Slit/Robo signaling regulates cell fate decisions in the intestinal stem cell lineage of *Drosophila*. Cell Rep. 7, 1867–1875. 10.1016/j.celrep.2014.05.024, PMID: 24931602 PMC4086754

[ref14] Capo F. Wilson A. Di Cara F. (2019). The intestine of *Drosophila melanogaster*: an emerging versatile model system to study intestinal epithelial homeostasis and host-microbial interactions in humans. Microorganisms 7:336. 10.3390/microorganisms7090336, PMID: 31505811 PMC6780840

[ref15] Charroux B. Capo F. Kurz C. L. Peslier S. Chaduli D. Viallat-Lieutaud A.. (2018). Cytosolic and secreted peptidoglycan-degrading enzymes in *Drosophila* respectively control local and systemic immune responses to microbiota. Cell Host Microbe 23, 215.e214–228.e214. 10.1016/j.chom.2017.12.007, PMID: 29398649

[ref16] Chen J. Kim S. M. Kwon J. Y. (2016a). A systematic analysis of *Drosophila* regulatory peptide expression in enteroendocrine cells. Mol. Cell 39, 358–366. 10.14348/molcells.2016.0014, PMID: 27025390 PMC4844944

[ref17] Chen J. Reiher W. Hermann-Luibl C. Sellami A. Cognigni P. Kondo S.. (2016b). Allatostatin A signalling in *Drosophila* regulates feeding and sleep and is modulated by PDF. PLoS Genet. 12:e1006346. 10.1371/journal.pgen.1006346, PMID: 27689358 PMC5045179

[ref18] Chintapalli V. R. Terhzaz S. Wang J. Al Bratty M. Watson D. G. Herzyk P.. (2012). Functional correlates of positional and gender-specific renal asymmetry in *Drosophila*. PLoS One 7:e32577. 10.1371/journal.pone.0032577, PMID: 22496733 PMC3319558

[ref19] Chng W. B. Bou Sleiman M. S. Schupfer F. Lemaitre B. (2014). Transforming growth factor beta/activin signaling functions as a sugar-sensing feedback loop to regulate digestive enzyme expression. Cell Rep. 9, 336–348. 10.1016/j.celrep.2014.08.064, PMID: 25284780

[ref20] Chtarbanova S. Lamiable O. Lee K. Z. Galiana D. Troxler L. Meignin C.. (2014). *Drosophila* C virus systemic infection leads to intestinal obstruction. J. Virol. 88, 14057–14069. 10.1128/JVI.02320-14, PMID: 25253354 PMC4249126

[ref21] Coast G. M. Webster S. G. Schegg K. M. Tobe S. S. Schooley D. A. (2001). The *Drosophila melanogaster* homologue of an insect calcitonin-like diuretic peptide stimulates V-ATPase activity in fruit fly Malpighian tubules. J. Exp. Biol. 204, 1795–1804. PMID: 11316500 10.1242/jeb.204.10.1795

[ref22] Dai X. Zhou E. Yang W. Zhang X. Zhang W. Rao Y. (2019). D-serine made by serine racemase in *Drosophila* intestine plays a physiological role in sleep. Nat. Commun. 10:1986. 10.1038/s41467-019-09544-9, PMID: 31064979 PMC6504911

[ref23] Depetris-Chauvin A. Galagovsky D. Chevalier C. Maniere G. Grosjean Y. (2017). Olfactory detection of a bacterial short-chain fatty acid acts as an orexigenic signal in *Drosophila melanogaster* larvae. Sci. Rep. 7:14230. 10.1038/s41598-017-14589-1, PMID: 29079812 PMC5660182

[ref24] Doupe D. P. Marshall O. J. Dayton H. Brand A. H. Perrimon N. (2018). *Drosophila* intestinal stem and progenitor cells are major sources and regulators of homeostatic niche signals. Proc. Natl. Acad. Sci. U. S. A. 115, 12218–12223. 10.1073/pnas.1719169115, PMID: 30404917 PMC6275525

[ref25] Droujinine I. A. Wang D. Hu Y. Udeshi D. Mu L. Svinkina T. (2020). Proteomics of protein trafficking by *in vivo* tissue-specific labeling. bioRxiv [Preprint]. 10.1101/2020.04.15.039933 PMC8062696 33888706

[ref26] Du E. J. Ahn T. J. Kwon I. Lee J. H. Park J. H. Park S. H.. (2016). TrpA1 regulates defecation of food-borne pathogens under the control of the Duox pathway. PLoS Genet. 12:e1005773. 10.1371/journal.pgen.1005773, PMID: 26726767 PMC4699737

[ref27] Dus M. Lai J. S. Gunapala K. M. Min S. Tayler T. D. Hergarden A. C.. (2015). Nutrient sensor in the brain directs the action of the brain-gut axis in *Drosophila*. Neuron 87, 139–151. 10.1016/j.neuron.2015.05.032, PMID: 26074004 PMC4697866

[ref28] Ghosh A. C. O'Connor M. B. (2014). Systemic Activin signaling independently regulates sugar homeostasis, cellular metabolism, and pH balance in *Drosophila melanogaster*. Proc. Natl. Acad. Sci. U. S. A. 111, 5729–5734. 10.1073/pnas.1319116111, PMID: 24706779 PMC3992655

[ref29] Ghosh A. Tattikota S. Liu Y. Comjean A. Hu Y. Barrera V. (2019). Drosophila PDGF/VEGF signaling from muscles to hepatocyte-like cells protects against obesity. bioRxiv [Preprint]. 10.1101/2019.12.23.887059 PMC7752135 33107824

[ref30] Gronke S. Muller G. Hirsch J. Fellert S. Andreou A. Haase T.. (2007). Dual lipolytic control of body fat storage and mobilization in *Drosophila*. PLoS Biol. 5:e137. 10.1371/journal.pbio.0050137, PMID: 17488184 PMC1865564

[ref31] Guo L. Karpac J. Tran S. L. Jasper H. (2014). PGRP-SC2 promotes gut immune homeostasis to limit commensal dysbiosis and extend lifespan. Cell 156, 109–122. 10.1016/j.cell.2013.12.018, PMID: 24439372 PMC3928474

[ref32] Guo X. Yin C. Yang F. Zhang Y. Huang H. Wang J.. (2019). The cellular diversity and transcription factor code of *Drosophila* enteroendocrine cells. Cell Rep. 29, 4172.e4175–4185.e4175. 10.1016/j.celrep.2019.11.048, PMID: 31851941

[ref33] Ha E. M. Oh C. T. Bae Y. S. Lee W. J. (2005). A direct role for dual oxidase in *Drosophila* gut immunity. Science 310, 847–850. 10.1126/science.1117311, PMID: 16272120

[ref34] Hang S. Purdy A. E. Robins W. P. Wang Z. Mandal M. Chang S.. (2014). The acetate switch of an intestinal pathogen disrupts host insulin signaling and lipid metabolism. Cell Host Microbe 16, 592–604. 10.1016/j.chom.2014.10.006, PMID: 25525791 PMC4272434

[ref35] Hanlon C. D. Andrew D. J. (2015). Outside-in signaling—a brief review of GPCR signaling with a focus on the *Drosophila* GPCR family. J. Cell Sci. 128, 3533–3542. 10.1242/jcs.175158, PMID: 26345366 PMC4610211

[ref36] Harsh S. Heryanto C. Eleftherianos I. (2019). Intestinal lipid droplets as novel mediators of host-pathogen interaction in *Drosophila*. Biol. Open 8:bio039040. 10.1242/bio.039040, PMID: 31278163 PMC6679391

[ref37] He L. Si G. Huang J. Samuel A. D. T. Perrimon N. (2018). Mechanical regulation of stem-cell differentiation by the stretch-activated Piezo channel. Nature 555, 103–106. 10.1038/nature25744, PMID: 29414942 PMC6101000

[ref38] Hentze J. L. Carlsson M. A. Kondo S. Nassel D. R. Rewitz K. F. (2015). The neuropeptide allatostatin A regulates metabolism and feeding decisions in *Drosophila*. Sci. Rep. 5:11680. 10.1038/srep11680, PMID: 26123697 PMC4485031

[ref39] Hergarden A. C. Tayler T. D. Anderson D. J. (2012). Allatostatin-A neurons inhibit feeding behavior in adult *Drosophila*. Proc. Natl. Acad. Sci. U. S. A. 109, 3967–3972. 10.1073/pnas.1200778109, PMID: 22345563 PMC3309792

[ref40] Honegger B. Galic M. Kohler K. Wittwer F. Brogiolo W. Hafen E.. (2008). Imp-L2, a putative homolog of vertebrate IGF-binding protein 7, counteracts insulin signaling in *Drosophila* and is essential for starvation resistance. J. Biol. 7:10. 10.1186/jbiol72, PMID: 18412985 PMC2323038

[ref41] Huang R. Song T. Su H. Lai Z. Qin W. Tian Y.. (2020). High-fat diet enhances starvation-induced hyperactivity via sensitizing hunger-sensing neurons in *Drosophila*. eLife 9:e53103. 10.7554/eLife.53103, PMID: 32324135 PMC7274782

[ref42] Hung R. J. Hu Y. Kirchner R. Liu Y. Xu C. Comjean A.. (2020). A cell atlas of the adult *Drosophila* midgut. Proc. Natl. Acad. Sci. U. S. A. 117, 1514–1523. 10.1073/pnas.1916820117, PMID: 31915294 PMC6983450

[ref43] Iatsenko I. Kondo S. Mengin-Lecreulx D. Lemaitre B. (2016). PGRP-SD, an extracellular pattern-recognition receptor, enhances peptidoglycan-mediated activation of the *Drosophila* Imd pathway. Immunity 45, 1013–1023. 10.1016/j.immuni.2016.10.029, PMID: 27851910

[ref44] Jiang H. Edgar B. A. (2009). EGFR signaling regulates the proliferation of *Drosophila* adult midgut progenitors. Development 136, 483–493. 10.1242/dev.026955, PMID: 19141677 PMC2687592

[ref45] Jiang H. Grenley M. O. Bravo M. J. Blumhagen R. Z. Edgar B. A. (2011). EGFR/Ras/MAPK signaling mediates adult midgut epithelial homeostasis and regeneration in *Drosophila*. Cell Stem Cell 8, 84–95. 10.1016/j.stem.2010.11.026, PMID: 21167805 PMC3021119

[ref46] Jiang H. Patel P. H. Kohlmaier A. Grenley M. O. McEwen D. G. Edgar B. A. (2009). Cytokine/Jak/Stat signaling mediates regeneration and homeostasis in the *Drosophila* midgut. Cell 137, 1343–1355. 10.1016/j.cell.2009.05.014, PMID: 19563763 PMC2753793

[ref47] Johnson E. C. Shafer O. T. Trigg J. S. Park J. Schooley D. A. Dow J. A.. (2005). A novel diuretic hormone receptor in *Drosophila*: evidence for conservation of CGRP signaling. J. Exp. Biol. 208, 1239–1246. 10.1242/jeb.01529, PMID: 15781884

[ref48] Kamareddine L. Robins W. P. Berkey C. D. Mekalanos J. J. Watnick P. I. (2018). The *Drosophila* immune deficiency pathway modulates enteroendocrine function and host metabolism. Cell Metab. 28, 449.e445–462.e445. 10.1016/j.cmet.2018.05.026, PMID: 29937377 PMC6125180

[ref49] Karpac J. Biteau B. Jasper H. (2013). Misregulation of an adaptive metabolic response contributes to the age-related disruption of lipid homeostasis in *Drosophila*. Cell Rep. 4, 1250–1261. 10.1016/j.celrep.2013.08.004, PMID: 24035390 PMC3832190

[ref50] Karpowicz P. Perez J. Perrimon N. (2010). The Hippo tumor suppressor pathway regulates intestinal stem cell regeneration. Development 137, 4135–4145. 10.1242/dev.060483, PMID: 21098564 PMC2990205

[ref51] Kim S. K. Rulifson E. J. (2004). Conserved mechanisms of glucose sensing and regulation by *Drosophila* corpora cardiaca cells. Nature 431, 316–320. 10.1038/nature02897, PMID: 15372035

[ref52] Kockel L. Huq L. M. Ayyar A. Herold E. MacAlpine E. Logan M.. (2016). A *Drosophila* LexA enhancer-trap resource for developmental biology and neuroendocrine research. G3 6, 3017–3026. 10.1534/g3.116.031229, PMID: 27527793 PMC5068927

[ref53] Krejcova G. Danielova A. Nedbalova P. Kazek M. Strych L. Chawla G.. (2019). *Drosophila macrophages* switch to aerobic glycolysis to mount effective antibacterial defense. eLife 8:e50414. 10.7554/eLife.50414, PMID: 31609200 PMC6867711

[ref54] Kwon Y. Song W. Droujinine I. A. Hu Y. Asara J. M. Perrimon N. (2015). Systemic organ wasting induced by localized expression of the secreted insulin/IGF antagonist ImpL2. Dev. Cell 33, 36–46. 10.1016/j.devcel.2015.02.012, PMID: 25850671 PMC4437243

[ref55] LaJeunesse D. R. Johnson B. Presnell J. S. Catignas K. K. Zapotoczny G. (2010). Peristalsis in the junction region of the *Drosophila* larval midgut is modulated by DH31 expressing enteroendocrine cells. BMC Physiol. 10:14. 10.1186/1472-6793-10-14, PMID: 20698983 PMC2933646

[ref56] Lee K. A. Kim B. Bhin J. Kim D. H. You H. Kim E. K.. (2015). Bacterial uracil modulates *Drosophila* DUOX-dependent gut immunity via hedgehog-induced signaling endosomes. Cell Host Microbe 17, 191–204. 10.1016/j.chom.2014.12.012, PMID: 25639794

[ref57] Li Q. Nirala N. K. Nie Y. Chen H. J. Ostroff G. Mao J.. (2018a). Ingestion of food particles regulates the Mechanosensing Misshapen-Yorkie Pathway in *Drosophila* intestinal growth. Dev. Cell 45, 433.e436–449.e436. 10.1016/j.devcel.2018.04.014, PMID: 29754801 PMC7480018

[ref58] Li Z. Quan G. Jiang X. Yang Y. Ding X. Zhang D.. (2018b). Effects of metabolites derived from gut microbiota and hosts on pathogens. Front. Cell. Infect. Microbiol. 8:314. 10.3389/fcimb.2018.00314, PMID: 30276161 PMC6152485

[ref59] Li Z. Zhang Y. Han L. Shi L. Lin X. (2013). Trachea-derived dpp controls adult midgut homeostasis in *Drosophila*. Dev. Cell 24, 133–143. 10.1016/j.devcel.2012.12.010, PMID: 23369712

[ref60] Liu Q. Jin L. H. (2017). Organ-to-organ communication: a *Drosophila* gastrointestinal tract perspective. Front. Cell Dev. Biol. 5:29. 10.3389/fcell.2017.00029, PMID: 28421183 PMC5376570

[ref61] Luis N. M. Wang L. Ortega M. Deng H. Katewa S. D. Li P. W.. (2016). Intestinal IRE1 is required for increased triglyceride metabolism and longer lifespan under dietary restriction. Cell Rep. 17, 1207–1216. 10.1016/j.celrep.2016.10.003, PMID: 27783936 PMC5089850

[ref62] Maniere G. Alves G. Berthelot-Grosjean M. Grosjean Y. (2020). Growth regulation by amino acid transporters in *Drosophila* larvae. Cell. Mol. Life Sci. 10.1007/s00018-020-03535-6, PMID: [Epub ahead of print] 32358623 PMC7588360

[ref63] Martin J. L. Sanders E. N. Moreno-Roman P. Jaramillo Koyama L. A. Balachandra S. Du X.. (2018). Long-term live imaging of the *Drosophila* adult midgut reveals real-time dynamics of division, differentiation and loss. eLife 7:e36248. 10.7554/eLife.36248, PMID: 30427308 PMC6277200

[ref64] Martin A. M. Sun E. W. Keating D. J. (2019). Mechanisms controlling hormone secretion in human gut and its relevance to metabolism. J. Endocrinol. 244, R1–R15. 10.1530/JOE-19-0399, PMID: 31751295 PMC6892457

[ref65] Miguel-Aliaga I. Jasper H. Lemaitre B. (2018). Anatomy and physiology of the digestive tract of *Drosophila melanogaster*. Genetics 210, 357–396. 10.1534/genetics.118.300224, PMID: 30287514 PMC6216580

[ref66] Obniski R. Sieber M. Spradling A. C. (2018). Dietary lipids modulate notch signaling and influence adult intestinal development and metabolism in *Drosophila*. Dev. Cell 47, 98.e115–111.e115. 10.1016/j.devcel.2018.08.013, PMID: 30220569 PMC6894183

[ref67] O’Brien L. E. Soliman S. S. Li X. Bilder D. (2011). Altered modes of stem cell division drive adaptive intestinal growth. Cell 147, 603–614. 10.1016/j.cell.2011.08.048, PMID: 22036568 PMC3246009

[ref68] Oh Y. Lai J. S. Mills H. J. Erdjument-Bromage H. Giammarinaro B. Saadipour K.. (2019). A glucose-sensing neuron pair regulates insulin and glucagon in *Drosophila*. Nature 574, 559–564. 10.1038/s41586-019-1675-4, PMID: 31645735 PMC6857815

[ref69] Ohlstein B. Spradling A. (2007). Multipotent *Drosophila* intestinal stem cells specify daughter cell fates by differential notch signaling. Science 315, 988–992. 10.1126/science.1136606, PMID: 17303754

[ref70] Osman D. Buchon N. Chakrabarti S. Huang Y. T. Su W. C. Poidevin M.. (2012). Autocrine and paracrine unpaired signaling regulate intestinal stem cell maintenance and division. J. Cell Sci. 125, 5944–5949. 10.1242/jcs.113100, PMID: 23038775

[ref71] Park J. H. Chen J. Jang S. Ahn T. J. Kang K. Choi M. S.. (2016). A subset of enteroendocrine cells is activated by amino acids in the *Drosophila* midgut. FEBS Lett. 590, 493–500. 10.1002/1873-3468.12073, PMID: 26801353

[ref72] Park J. H. Kwon J. Y. (2011). Heterogeneous expression of *Drosophila* gustatory receptors in enteroendocrine cells. PLoS One 6:e29022. 10.1371/journal.pone.0029022, PMID: 22194978 PMC3237578

[ref73] Poels J. Birse R. T. Nachman R. J. Fichna J. Janecka A. Vanden Broeck J.. (2009). Characterization and distribution of NKD, a receptor for *Drosophila* tachykinin-related peptide 6. Peptides 30, 545–556. 10.1016/j.peptides.2008.10.012, PMID: 19022310

[ref74] Qiao H. Keesey I. W. Hansson B. S. Knaden M. (2019). Gut microbiota affects development and olfactory behavior in *Drosophila melanogaster*. J. Exp. Biol. 222:jeb192500. 10.1242/jeb.192500, PMID: 30679242

[ref75] Reiher W. Shirras C. Kahnt J. Baumeister S. Isaac R. E. Wegener C. (2011). Peptidomics and peptide hormone processing in the *Drosophila* midgut. J. Proteome Res. 10, 1881–1892. 10.1021/pr101116g, PMID: 21214272

[ref76] Ren G. R. Hauser F. Rewitz K. F. Kondo S. Engelbrecht A. F. Didriksen A. K.. (2015). CCHamide-2 is an orexigenic brain-gut peptide in *Drosophila*. PLoS One 10:e0133017. 10.1371/journal.pone.0133017, PMID: 26168160 PMC4500396

[ref77] Riabinina O. Potter C. J. (2016). The Q-system: a versatile expression system for *Drosophila*. Methods Mol. Biol. 1478, 53–78. 10.1007/978-1-4939-6371-3_3, PMID: 27730575 PMC5270762

[ref78] Rodenfels J. Lavrynenko O. Ayciriex S. Sampaio J. L. Carvalho M. Shevchenko A.. (2014). Production of systemically circulating hedgehog by the intestine couples nutrition to growth and development. Genes Dev. 28, 2636–2651. 10.1101/gad.249763.114, PMID: 25452274 PMC4248294

[ref79] Rommelaere S. Boquete J. P. Piton J. Kondo S. Lemaitre B. (2019). The exchangeable apolipoprotein Nplp2 sustains lipid flow and heat acclimation in *Drosophila*. Cell Rep. 27, 886.e886–899.e886. 10.1016/j.celrep.2019.03.074, PMID: 30995484

[ref80] Royet J. Charroux B. (2013). Mechanisms and consequence of bacteria detection by the *Drosophila* gut epithelium. Gut Microbes 4, 259–263. 10.4161/gmic.24386, PMID: 23633672 PMC3669173

[ref81] Sano H. Nakamura A. Texada M. J. Truman J. W. Ishimoto H. Kamikouchi A.. (2015). The nutrient-responsive hormone CCHamide-2 controls growth by regulating insulin-like peptides in the brain of *Drosophila melanogaster*. PLoS Genet. 11:e1005209. 10.1371/journal.pgen.1005209, PMID: 26020940 PMC4447355

[ref82] Sansone C. L. Cohen J. Yasunaga A. Xu J. Osborn G. Subramanian H.. (2015). Microbiota-dependent priming of antiviral intestinal immunity in *Drosophila*. Cell Host Microbe 18, 571–581. 10.1016/j.chom.2015.10.010, PMID: 26567510 PMC4648705

[ref83] Schmidt P. T. Tornoe K. Poulsen S. S. Rasmussen T. N. Holst J. J. (2000). Tachykinins in the porcine pancreas: potent exocrine and endocrine effects via NK-1 receptors. Pancreas 20, 241–247. 10.1097/00006676-200004000-00004, PMID: 10766449

[ref84] Scopelliti A. Bauer C. Yu Y. Zhang T. Kruspig B. Murphy D. J.. (2019). A neuronal relay mediates a nutrient responsive gut/fat body axis regulating energy homeostasis in adult *Drosophila*. Cell Metab. 29:269.e210–284.e210. 10.1016/j.cmet.2018.09.021, PMID: 30344016 PMC6370946

[ref85] Shen R. Wang B. Giribaldi M. G. Ayres J. Thomas J. B. Montminy M. (2016). Neuronal energy-sensing pathway promotes energy balance by modulating disease tolerance. Proc. Natl. Acad. Sci. U. S. A. 113, E3307–E3314. 10.1073/pnas.1606106113, PMID: 27208092 PMC4988575

[ref86] Shin S. C. Kim S. H. You H. Kim B. Kim A. C. Lee K. A.. (2011). *Drosophila* microbiome modulates host developmental and metabolic homeostasis via insulin signaling. Science 334, 670–674. 10.1126/science.1212782, PMID: 22053049

[ref87] Siviter R. J. Coast G. M. Winther A. M. Nachman R. J. Taylor C. A. Shirras A. D.. (2000). Expression and functional characterization of a *Drosophila* neuropeptide precursor with homology to mammalian preprotachykinin A. J. Biol. Chem. 275, 23273–23280. 10.1074/jbc.M002875200, PMID: 10801863

[ref88] Soderberg J. A. Birse R. T. Nassel D. R. (2011). Insulin production and signaling in renal tubules of *Drosophila* is under control of tachykinin-related peptide and regulates stress resistance. PLoS One 6:e19866. 10.1371/journal.pone.0019866, PMID: 21572965 PMC3091884

[ref89] Song W. Cheng D. Hong S. Sappe B. Hu Y. Wei N.. (2017). Midgut-derived activin regulates glucagon-like action in the fat body and glycemic control. Cell Metab. 25, 386–399. 10.1016/j.cmet.2017.01.002, PMID: 28178568 PMC5373560

[ref90] Song W. Kir S. Hong S. Hu Y. Wang X. Binari R.. (2019). Tumor-derived ligands trigger tumor growth and host wasting via differential MEK activation. Dev. Cell 48, 277.e6–286.e6. 10.1016/j.devcel.2018.12.003, PMID: 30639055 PMC6368352

[ref91] Song W. Ren D. Li W. Jiang L. Cho K. W. Huang P.. (2010). SH2B regulation of growth, metabolism, and longevity in both insects and mammals. Cell Metab. 11, 427–437. 10.1016/j.cmet.2010.04.002, PMID: 20417156 PMC2881875

[ref92] Song W. Veenstra J. A. Perrimon N. (2014). Control of lipid metabolism by tachykinin in *Drosophila*. Cell Rep. 9, 40–47. 10.1016/j.celrep.2014.08.060, PMID: 25263556 PMC4325997

[ref93] Stahl B. A. Slocumb M. E. Chaitin H. DiAngelo J. R. Keene A. C. (2017). Sleep-dependent modulation of metabolic rate in *Drosophila*. Sleep 40:zsx084. 10.1093/sleep/zsx084, PMID: 28541527 PMC6074949

[ref94] Stevens L. M. Zhang Y. Volnov Y. Chen G. Stein D. S. (2019). Isolation of secreted proteins from *Drosophila* ovaries and embryos through in vivo BirA-mediated biotinylation. PLoS One 14:e0219878. 10.1371/journal.pone.0219878, PMID: 31658274 PMC6816556

[ref95] Storelli G. Defaye A. Erkosar B. Hols P. Royet J. Leulier F. (2011). *Lactobacillus plantarum* promotes *Drosophila* systemic growth by modulating hormonal signals through TOR-dependent nutrient sensing. Cell Metab. 14, 403–414. 10.1016/j.cmet.2011.07.012, PMID: 21907145

[ref96] Sun E. W. L. Martin A. M. Young R. L. Keating D. J. (2018). The regulation of peripheral metabolism by gut-derived hormones. Front. Endocrinol. 9:754. 10.3389/fendo.2018.00754, PMID: 30662430 PMC6328484

[ref97] Sun K. Wernstedt Asterholm I. Kusminski C. M. Bueno A. C. Wang Z. V. Pollard J. W.. (2012). Dichotomous effects of VEGF-A on adipose tissue dysfunction. Proc. Natl. Acad. Sci. U. S. A. 109, 5874–5879. 10.1073/pnas.1200447109, PMID: 22451920 PMC3326476

[ref98] Takeda K. Okumura T. Terahata M. Yamaguchi M. Taniguchi K. Adachi-Yamada T. (2018). *Drosophila* peptide hormones allatostatin A and diuretic hormone 31 exhibiting complementary gradient distribution in posterior midgut antagonistically regulate midgut senescence and adult lifespan. Zool. Sci. 35, 75–85. 10.2108/zs160210, PMID: 29417892

[ref99] Talsma A. D. Christov C. P. Terriente-Felix A. Linneweber G. A. Perea D. Wayland M.. (2012). Remote control of renal physiology by the intestinal neuropeptide pigment-dispersing factor in *Drosophila*. Proc. Natl. Acad. Sci. U. S. A. 109, 12177–12182. 10.1073/pnas.1200247109, PMID: 22778427 PMC3409758

[ref100] Tang G. Wang Y. Park S. Bajpayee N. S. Vi D. Nagaoka Y.. (2012). Go2 G protein mediates galanin inhibitory effects on insulin release from pancreatic beta cells. Proc. Natl. Acad. Sci. U. S. A. 109, 2636–2641. 10.1073/pnas.1200100109, PMID: 22308501 PMC3289306

[ref101] Teperino R. Aberger F. Esterbauer H. Riobo N. Pospisilik J. A. (2014). Canonical and non-canonical Hedgehog signalling and the control of metabolism. Semin. Cell Dev. Biol. 33, 81–92. 10.1016/j.semcdb.2014.05.007, PMID: 24862854 PMC4130743

[ref102] Tian A. Benchabane H. Wang Z. Ahmed Y. (2016). Regulation of stem cell proliferation and cell fate specification by Wingless/Wnt signaling gradients enriched at adult intestinal compartment boundaries. PLoS Genet. 12:e1005822. 10.1371/journal.pgen.1005822, PMID: 26845150 PMC4742051

[ref103] Tian A. Jiang J. (2014). Intestinal epithelium-derived BMP controls stem cell self-renewal in *Drosophila* adult midgut. eLife 3:e01857. 10.7554/eLife.01857, PMID: 24618900 PMC3948108

[ref104] Tian A. Jiang J. (2017). Dual role of BMP signaling in the regulation of *Drosophila* intestinal stem cell self-renewal. Fly 11, 297–302. 10.1080/19336934.2017.1384104, PMID: 28945500 PMC5721945

[ref105] Tian A. Shi Q. Jiang A. Li S. Wang B. Jiang J. (2015). Injury-stimulated hedgehog signaling promotes regenerative proliferation of *Drosophila* intestinal stem cells. J. Cell Biol. 208, 807–819. 10.1083/jcb.201409025, PMID: 25753035 PMC4362464

[ref106] Vaccaro A. Kaplan Dor Y. Nambara K. Pollina E. A. Lin C. Greenberg M. E.. (2020). Sleep loss can cause death through accumulation of reactive oxygen species in the gut. Cell 181, 1307.e1315–1328.e1315. 10.1016/j.cell.2020.04.049, PMID: 32502393

[ref107] Valzania L. Coon K. L. Vogel K. J. Brown M. R. Strand M. R. (2018). Hypoxia-induced transcription factor signaling is essential for larval growth of the mosquito *Aedes aegypti*. Proc. Natl. Acad. Sci. U. S. A. 115, 457–465. 10.1073/pnas.1719063115, PMID: 29298915 PMC5777003

[ref108] Veenstra J. A. (2009). Peptidergic paracrine and endocrine cells in the midgut of the fruit fly maggot. Cell Tissue Res. 336, 309–323. 10.1007/s00441-009-0769-y, PMID: 19319573

[ref109] Veenstra J. A. Agricola H. J. Sellami A. (2008). Regulatory peptides in fruit fly midgut. Cell Tissue Res. 334, 499–516. 10.1007/s00441-008-0708-3, PMID: 18972134

[ref110] Veenstra J. A. Ida T. (2014). More *Drosophila* enteroendocrine peptides: Orcokinin B and the CCHamides 1 and 2. Cell Tissue Res. 357, 607–621. 10.1007/s00441-014-1880-2, PMID: 24850274

[ref111] Werner T. Liu G. Kang D. Ekengren S. Steiner H. Hultmark D. (2000). A family of peptidoglycan recognition proteins in the fruit fly *Drosophila melanogaster*. Proc. Natl. Acad. Sci. U. S. A. 97, 13772–13777. 10.1073/pnas.97.25.13772, PMID: 11106397 PMC17651

[ref112] Winther A. M. Nassel D. R. (2001). Intestinal peptides as circulating hormones: release of tachykinin-related peptide from the locust and cockroach midgut. J. Exp. Biol. 204, 1269–1280. PMID: 11249837 10.1242/jeb.204.7.1269

[ref113] Wong A. C. Dobson A. J. Douglas A. E. (2014). Gut microbiota dictates the metabolic response of *Drosophila* to diet. J. Exp. Biol. 217, 1894–1901. 10.1242/jeb.101725, PMID: 24577449 PMC4037322

[ref114] Xu C. Luo J. He L. Montell C. Perrimon N. (2017). Oxidative stress induces stem cell proliferation via TRPA1/RyR-mediated Ca(2+) signaling in the *Drosophila* midgut. eLife 6:e22441. 10.7554/eLife.22441, PMID: 28561738 PMC5451214

[ref115] Zhang J. Liu Y. Jiang K. Jia J. (2020). Hedgehog signaling promotes lipolysis in adipose tissue through directly regulating Bmm/ATGL lipase. Dev. Biol. 457, 128–139. 10.1016/j.ydbio.2019.09.009, PMID: 31550483 PMC7322621

[ref116] Zheng H. Wang X. Guo P. Ge W. Yan Q. Gao W.. (2017). Premature remodeling of fat body and fat mobilization triggered by platelet-derived growth factor/VEGF receptor in *Drosophila*. FASEB J. 31, 1964–1975. 10.1096/fj.201601127R, PMID: 28126734

